# Intra-dermal immunisation with SIV gag-based vaccines alone inhibits acquisition of SIVmac251

**DOI:** 10.1186/1742-4690-9-S2-O49

**Published:** 2012-09-13

**Authors:** N Almond, R Stebbings, M Page, B Li, N Berry, C Ham, D Ferguson, N Rose, E Mee, C Stahl-Hennig, G Dickson, T Athanasapoulos, A Benlahrech, S Herath, A Meiser, S Patterson

**Affiliations:** 1 NIBSC-HPA, Potters Bar, Hertfordshire, UK; 2 DPZ, Göttingen, Germany; 3 Royal Holloway, University of London, London, UK; 4 Imperial College, London, UK

## Background

It is increasingly recognised that effective anti-viral cell mediated immunity depends not only on the frequency of antigen specific T cells, but also on the quality of these T cells. We have evaluated a vaccine protocol involving DNA prime and Adenoviral vector boost to deliver SIV gag in MHC typed Mauritian derived Macaca fascicularis.

## Methods

Groups of 8 macaques were immunised 3 times on weeks 0, 4, 8 with 100μg purified plasmid DNA designed to express SIVmac239 derived gag under the control of the CMV immediate early promoter. Three different SIV gag vaccines were compared. The DNA plasmids expressed native SIVmac239gag, ubiquinated SIVmac239 gag or fragmented, ubiquinated SIVmac239 gag derived peptides. On week 19, the groups were boosted with 10e7 infectious units recombinant Ad 5 expressing the same SIV Gag antigens. Cell mediated immunity was assessed after each immunisation and after virus challenge. At week 23, all vaccinated macaques, along with a group of naive challenge controls began 10 weekly, atraumatic challenges via the rectal mucosal with 150TCD_50_ SIVmac251.

## Results

Delivery of this vaccine via the intra-dermal route elicited CD8 and CD4 T cell responses. Moreover, approximately 50% of antigen specific CD4+ T cells expressed the mucosal homing marker alpha4 beta7. When vaccinated macaques were exposed to a stock of uncloned SIVmac251 that had been propagated on simian PBMC’s, by repeated low dose challenge via the intra-rectal route, a significant delay (Wilcoxon test; p=0.015) in acquisition of SIV was obtained amongst vaccinated macaques compared with naive controls challenged in a similar manner. Furthermore, peak viral loads amongst vaccinated macaques were significantly lower than challenge controls (Kruskal-Wallis test; p=0.010).

## Conclusion

We are investigating whether the route of immunisation was crucial to the vaccine’s success.

